# Stress-Related Sleep Disorders and Their Impact On Adolescents’ Physical Health

**DOI:** 10.1192/j.eurpsy.2026.12091

**Published:** 2026-07-31

**Authors:** M. Rmili, S. Boulbaroud, M. El Ayachi, A. Chaker Lamrani, F.-Z. Azzaoui

**Affiliations:** 1 Laboratory of Biology and Health, Faculty of Sciences, Kenitra, Morocco; 2 Unit of Biotechnology and Sustainable Development of Natural Resources, Polydisciplinary Faculty, Sultan My. Slimane University, Beni Mellal, Morocco

## Abstract

**Introduction:**

Many young people experience sleep disorders, which may be linked to multiple factors. Poor sleep quality can have negative effects on adolescents’ physical health.

**Objectives:**

To examine the relationship between physical health and stress-related sleep disorders.

**Methods:**

This cross-sectional study with an analytical aims, was conducted at the Oued Eddahab High School in the city of Tiflet (The Rabat-Salé-Kenitra Region). The study included 378 students (239 girls and 139 boys) with a mean age of 17.08 ± 1.28 years. Participants completed a questionnaire that collected data on age, sex, back pain, headaches, neck, shoulder, and hand pains, as well as sleep disorders. The 10-item Perceived Stress Scale (PSS-10) was also used to assess their stress levels.

**Results:**

39.4% of the participants suffered from sleep disorders. There was a highly significant difference in sleep disorders between girls and boys (χ^2^ = 9.062 ; p<0.01). Among girls, perceived stress showed a highly significant association with sleep disorders (χ^2^ = 10.418 ; p<0.01) and a significant association among boys (χ^2^ = 6.771 ; p<0.05). Sleep disorders showed a highly significant association with headaches, neck, shoulder, and hand pains among girls (χ^2^ = 9.325 ; p<0.01) and among boys (χ^2^ = 11.353 ; p<0.01). Sleep disorders showed a highly significant association with back pain only among boys (χ^2^ = 9.429 ; p<0.01).

**Conclusions:**

Sleep disorders result from perceived stress. These disorders can cause headaches, neck, shoulder, and hand pain in both sexes, and back pain only in boys.

**Disclosure of Interest:**

None Declared

